# Survey on Psychological Well-Being and Quality of Life in Visually Impaired Individuals: Dancesport vs. Other Sound Input-Based Sports

**DOI:** 10.3390/ijerph19084438

**Published:** 2022-04-07

**Authors:** Giuditta Carretti, Daniela Mirandola, Eleonora Sgambati, Mirko Manetti, Mirca Marini

**Affiliations:** 1 Section of Anatomy and Histology, Department of Experimental and Clinical Medicine, University of Florence, 50134 Florence, Italy; giuditta.carretti@unifi.it (G.C.); daniela.mirandola@unifi.it (D.M.); mirko.manetti@unifi.it (M.M.); 2 Department of Biosciences and Territory, University of Molise, 86090 Pesche, Italy; eleonora.sgambati@unimol.it

**Keywords:** visual disability, dancesport, sound input-based sport, quality of life, psychological well-being, survey

## Abstract

Sport practice has the widely demonstrated potential of promoting well-being and physical/mental health, especially in disabled individuals. Nowadays, visually impaired people can participate in several sports commonly adapted and played substituting visual input with auditory or tactile ones. By integrating movement and music, dance can simultaneously promote physical and emotional involvement and enhances vicarious sense recruitment. On these premises, we performed a survey to assess the psychological well-being (PWB) and quality of life (QoL) in visually impaired athletes, comparing dancesport vs other sound input-based sports. Twenty-one visually impaired dancers and twenty-seven visually impaired athletes practicing adapted baseball, showdown, blind futsal, or blind tennis completed a structured self-report survey including the Italian version of PWB-18 scale and the Short Form-12 (SF-12) questionnaire. Dancers reported significantly higher scores in PWB-18 autonomy, environmental mastery, and self-acceptance along with a higher PWB total score than the other athlete group. Similarly, the SF-12 questionnaire results demonstrated significantly higher scores in both physical and mental QoL of visually impaired dancers compared with other athletes. In conclusion, our findings suggest that, given its peculiarities, the practice of dancesport may have a stronger positive impact on PWB and QoL of visually impaired individuals than other sound input-based sports.

## 1. Introduction

Visual impairment, either congenital or acquired, isolates the subject from social context and reduces physical and recreational activity, thus inducing to a sedentary lifestyle [1,2]. It is widely demonstrated that a physically active lifestyle is essential for everyone, especially for people with disabilities, since it plays a key-role on health, quality of life (QoL) perception and physical/mental well-being of an individual [3,4,5,6,7,8,9]. Visual system enables the brain to assess and process information regarding the position of the body in the space, adjusting posture accordingly, and provides movement precision and motor reaction timing [10,11,12]. Consequently, a partial or total lack of sight leads to deficiencies in bilateral coordination [13,14] and posture due to inadequate interaction with the environment and affects gait patterns and balance [14,15,16]. Intact balance control is required not only for postural stability but also for safe mobility-related daily activities. Moreover, in order to compensate for faulty gait, postural alterations develop in visually impaired subjects thus increasing musculoskeletal disorders, pain syndromes and falls risk, hence generating and feeding a vicious circle [17,18,19,20,21]. Posture is an important health indicator and, hence, its alterations are associated with a large number of psychophysical disorders which lead to a consequently loss of autonomy, social integration, and QoL. Vision and audition represent the main senses recruited to explore and manage space and time dimensions and, specifically, the visual system is the most accurate tool for processing details and topographical aspects of the environment [22,23,24]. Despite this specialization, senses operate together to build perceptions through multisensory input integration. According to the sensory compensation hypothesis and owing to neural plasticity skill of the brain, sensory deprivation generates a functional enhancement of the remaining senses [10]. Indeed, visually deprived individuals firmly rely on auditory references to orient in space, and their spatial awareness/perception is based more on body-centered reference frames than on the external environment ones [25,26,27,28].

On this basis, most of the adapted sports for blind and visually impaired subjects are commonly conceived and played substituting visual input with a vicarious sensory channel, generally auditory or tactile [3,29]. Blind adapted sports such as baseball, futsal, tennis, and showdown exploit simple sonorous stimuli to allow players to properly localize the ball and direct/coordinate motor action. Conversely, dance is based on the natural integration of multimodal stimuli organized in rhythmic sequences, thus promoting both physical and emotional involvement and increasing the ability of the subject to perceive the multisensory nature of reality [30,31]. The term multimodal refers to stimuli that generate simultaneous information in more than one sensory channel. According to the intersensory redundancy hypothesis, amodal properties processing is promoted by multimodal stimulation that helps binding and integrating multisensory input. Another feature that appears to support this process is motion, especially when accompanied by a rhythmic sound [22,30]. In order to acquire and generate appropriately timed behavioral responses, temporal/spatial synchrony is known to be essential. By blending multisensory input, dance synchronizes movement, expression, and perception instead of using a unimodal signal to subsequently direct action, thus recalling the sensory-motor simultaneity of daily-life events [25,26,27,28]. Additionally, dance shifts the focus from mere physical performance to individual holistic expression promoting body language. Rhythmic bodily movements on music, simultaneously aesthetic and athletic, allow to experience the full spectrum of space dimensions and body movement possibilities, therefore increasing orientation skills and favoring the acquisition of a correct self-image [32,33,34]. Constant practice enhances breath control, coordination, balance, and proprioception thus improving motor efficiency, autonomy, and self-confidence [33,35,36]. Pointedly, dancesport is a partnered dance and this peculiarity implicates a high degree of personal involvement in social interaction, communication, emotional regulation, and tactile sensory activation, which counteracts feelings frequently related to disability such as loneliness, isolation, and anxiety [37]. 

Although there is growing acknowledgement of artistic involvement benefits to well-being and health promotion in the general population [38], almost no scientific research has addressed it to sight impaired individuals. Therefore, the present study employed a structured online survey with the aim to investigate the possible benefits of dancesport on psychological well-being (PWB) and QoL of visually impaired subjects by comparing this practice with other adapted sports based on simple/non rhythmic sound input.

## 2. Materials and Methods

### 2.1. Participants

The study participants consisted of 48 visually impaired athletes who answered a structured self-report survey to assess PWB and QoL. Specifically, 21 were visually impaired dancesport dancers, whereas 27 were visually impaired athletes practicing other sound input-based sports such as adapted blind baseball, showdown, blind futsal, and blind tennis. Training plan of different sports was as follows: baseball, 2.5 h session once a week; tennis, showdown and dancesport, 2 h session twice a week; and futsal, 1.5 h session twice a week. Concerning the athlete career and expertise, sport practice comprised between 6 months and a year corresponded to beginner, from 1 to 3 years to intermediate, and over 3 years to advanced level profile. On the basis of this classification, the different sport-specific samples were composed as follows: 10 advanced, 4 intermediate, and 7 beginner dancers; 10 advanced tennis players; 3 advanced, 1 intermediate, and 2 beginner baseball athletes; 6 advanced showdown players; and 2 advanced, 2 intermediate, and 1 beginner futsal players. Habitual and current practice of dancesport or other mentioned sport activities at the moment of filling out the questionnaire was set as inclusion criterion. Exclusion criteria were represented by any behavioral/mental disorder or chronic pathology. Study procedures were carried out following the rules of the Declaration of Helsinki of 1975 (https://www.wma.net/what-we-do/medical-ethics/declaration-ofhelsinki/; accessed on 16 September 2021), revised in 2013. All subjects participated voluntarily and anonymously and gave their informed consent. No ethics committee approval was needed for this anonymous online survey.

### 2.2. Online Survey

Data were collected employing an online survey questionnaire created through the Google Forms platform and distributed via e-mail [29,39,40], in the form of a shareable and direct access link, to visually impaired athletes using the mailing list of specific sport associations which they were registered to (i.e., “Se mi aiuti ballo anche io” and “Scuola di ballo MG” in Siena, “Balliamo insieme” in Rome, and “Semplicemente danza” in Savona for dancesport; “Quarto Tempo” in Florence for blind futsal; “Polisportiva Silvano Dani” in Florence for adapted blind baseball; “ASD Disabili Firenze” in Florence for showndown; and “Virtus Tennis” in Bologna for blind tennis). Each subject voluntarily took part in the study and answered to the survey using specific computer or cell phone assistive technology for blind or visually impaired individuals, as previously described [29]. The self-administered questionnaire responses were completely anonymous and confidential and recorded in the Google Survey database. The survey was structured in a first part collecting sociodemographic characteristics such as age, gender, and educational status, as well as information about the type and severity of visual disability and the age of sport-specific practice. The second part comprised the 18-item Italian version of the PWB (PWB-18) scale and the Italian version of Short Form-12 (SF-12) to assess the PWB and QoL, respectively [29,41,42,43,44,45]. In particular, the modified 18-item version of Ryff’s Scales of PWB includes 3 items for each of the 6 well-being distinct dimensions investigated, namely self-acceptance, autonomy, environmental mastery, purpose in life, positive relations with others, and personal growth [46,47]. For each dimension, a high score indicates that the respondent has a mastery of that area. Conversely, a low score shows that the respondent struggles to feel comfortable with that particular concept. The total score is the mean of the ratings, with a higher score relating to a greater well-being [41,42,43]. The SF-12 questionnaire is among the most widely used tools for assessing self-reported health-related QoL and consists of 12 items used to investigate physical and mental dimensions. Higher scores on these subscales are associated to a better QoL [44,48]. The English version of the administered questionnaire is available as supporting information (see Supplementary Materials).

### 2.3. Statistical Analysis

Data were entered using Microsoft Office Excel. All data are represented as mean ± standard error of the mean (SEM) or mean ± standard deviation (SD), or percentage. SPSS version 25.0 (Statistical Package for the Social Sciences, Chicago, IL, USA) was used for statistical analyses. Differences between two groups were analyzed by unpaired Student’s *t*-test or chi-square test as appropriate. Multiple linear regression model including sex, blindness (i.e., congenital or acquired), visual disability level, educational level, years of sport practice and the type of practiced sport (i.e., dancesport or other sports) as fixed predictor variables and scores of PWB or QoL as dependent variables was also performed. Values of *p* < 0.05 were considered statistically significant.

## 3. Results

Overall, 21 visually impaired dancers (10 males, 11 females; mean age 54.6 ± 13.9 years) and 27 visually impaired athletes (19 males, 8 females; mean age 47.4 ± 11.1 years) practicing other adapted sports (i.e., baseball, showdown, futsal, and tennis) took part in this study by answering the self-administered questionnaire. Data concerning the sociodemographic characteristics and information on visual disability of the study participants are detailed in Table 1. No significant difference between the two groups was observed for the different variables taken into account (Table 1). 

The comparison of score results of PWB-18 scale and SF-12 questionnaire between visually impaired dancers and other sport players are shown in Table 2. 

Concerning the PWB evaluation, visually impaired dancers showed higher PWB-18 mean values than other athletes. In particular, dancers reported higher statistically significant values for PWB-18 autonomy, environmental mastery and self-acceptance dimensions compared with other athletes (Table 2). In addition, PWB total score of dancers was significantly higher than that of the other group (Table 2). 

Data concerning the assessment of perceived health status using the SF-12 questionnaire are also displayed in Table 2. SF-12 analysis revealed that dancers had significantly higher scores for both physical and mental QoL compared with data from other athletes.

Furthermore, multiple linear regression analysis including the overall characteristics of visually impaired subjects was performed to identify factors that had a significant independent impact on PWB (Table 3) and QoL (Table 4 and Table 5).

The practiced sport was an independent predictor either of PWB (*p* = 0.026; Table 3) or physical and mental QoL (*p* = 0.003 and *p* = 0.024, respectively; Table 4 and Table 5). In contrast, other factors including age, type of blindness, visual disability level, educational level and years of sport practice were not associated with PWB or QoL. The educational level was only predictor of physical QoL (*p* = 0.006; Table 4).

## 4. Discussion

To our knowledge, the present investigation is the first to assess the PWB and QoL of visually impaired individuals practicing dancesport compared to other sound input-based sports. 

QoL and PWB are multidimensional broad concepts related to physical, mental, and social domains which are composite and relate one to each other, thus causing the complexity of their measurement [49,50]. In case of disabled subjects, they are even more complex constructs due to physiological and psychological implications of the specific impairment [3]. The widely demonstrated positive impact of regular physical activity on overall QoL and PWB mainly relates to physical functioning improvement, anxiety level/depression risk reduction, mood enhancement, body awareness acquisition, as well as emotional and social involvement [3,6].

In a previous study we reported, for the first time, the psychophysical benefits of adapted baseball practice in visually impaired individuals [29,45]. Starting from this evidence and the key-role of vicarious senses, this study deepens the impact of audio-motor training on this target population especially focusing on sound input features of different adapted sports and their fallout on global subject involvement. Since prehistoric times, the ability to dance was seen as an important factor of physical and social fitness by our ancestors who used it as an innate and powerful tool for expression, communication, social bonding, spiritual healing, and self-awareness [51,52,53]. In recent decades, researchers begun to study those effects empirically, often referring to dance as an art sport, thus highlighting its potential of both emotional and physical involvement. Dance provides the opportunity of exploring all body possibilities, hence learning to use it creatively, unconventionally, and globally [54]. Regardless of the specific style, dance practice positively affects motor performance, coordination, flexibility, static and dynamic balance [32,35]. Naturally comprising social interaction, physical exercise and sensory/emotional stimulation through music, dance allows people to experience the pleasure of moving despite disability-related limitations, consequently fostering adherence to physical activity and counteracting sedentariness [55]. In case of vision loss, path integration and spatial memory required for a safe and efficient environment navigation mostly depend on body-centered references, vestibular input about head accelerations as well as proprioceptive information about self-motion [25,28]. 

Considering the significant score that we observed for the dancer group in both autonomy and environmental mastery PWB domains, it might be speculated that dance, through rapid direction changing, stopping, landing, turns, and rhythmic synchronized movements, may help to develop complex motor skills and spatial awareness which are crucial for orientation, fall risk prevention and independent mobility of sight impaired individuals [2,33,45,56]. This assumption is further strengthened by the statistically significant findings of the applied multiple linear regression model revealing the role of sport-specific practice as independent predictor of both PWB and QoL. On this basis, it might be hypothesized that dancesport practice, given its peculiar sensory-motor simultaneity features and improving skills commonly compromised in case of visual impairment, has the potential to positively affect such dimensions in this target population. In addition, given its peculiarities of partnered dance, dancesport is particularly suited to blind subjects because it is strongly based on the sense of touch and close physical contact between dancers. Moreover, the presence of a partner is considered a motivating factor for practice adherence [3,54]. It has also been demonstrated that multisensory training, combining haptic and audio features, might support and enhance perceptual functions, learning process, cognitive mapping, and exploration of the unknown space in blind subjects [30,31,57]. Complex athletic and aesthetic tasks engaging the whole body and implying diversified cognitive, motor, and relational responses, together with the high variability of music texture, might be the main characteristics which could explain the herein reported higher effectiveness of dancesport on both physical and mental dimension of QoL of visually impaired individuals compared with other sound input-based sports [58]. The required focus on body perceptions, sensory awareness, and global subject expression typical of dancing has the potential to increase emotional connectedness/regulation and decrease perceived isolation [59,60], therefore positively affecting PWB self-acceptance dimension, as demonstrated by our statistically significant findings. Even though some studies demonstrated that musical texture provides additional timing information compared with metronome or simple sound stimuli, and that rhythmic auditory cues promote feedforward control that allows movement anticipation, quick motor planning, coordinated muscle activation and neural plasticity [61,62], there is a lack of interventions including participants affected by visual disability. In fact, visual impairment or blindness are often considered exclusion factors from physical activity protocols. Moreover, as shown by a recent systematic review, the majority of the studies addressing the sight impaired population had a duration of 12 weeks or less, thus limiting possible effects on outcomes that may take a longer time to change [2]. In this context, we believe that our findings help filling this gap through the analysis we have conducted, in a real-world setting, of the multidimensional benefits of dancesport practice on QoL and PWB of visually impaired subjects in comparison with other sound input-based sports. 

Although encouraging, our findings need to be interpreted within the context of self-report, survey-based assessments. However, it should be considered that health psychology research studies are mainly based on data collected through questionnaires or surveys [28]. Moreover, considering the cross-sectional nature of our study, further longitudinal studies are also required. Finally, the positive impact of dancesport practice on PWB and QoL of subjects with visual impairment will require confirmation in larger and independent populations.

## 5. Conclusions

In summary, our survey data on overall PWB and QoL revealed higher statistically significant scores in visually impaired dancers with respect to athletes practicing other sound input-based sports. The motor and expressive peculiarities of dance may largely explain such a positive fallout on PWB and QoL. Therefore, we believe that visually disabled subjects should be encouraged to experience dancesport. In perspective, we are confident that the results of our study will pave the way for further investigation, especially concerning different dance styles and music texture/rhythm, as well as the multimodal training methodology to apply in protocols tailored to this target population.

## Figures and Tables

**Table 1:** Demographic data and description of visual disability of visually impaired subjects practicing dancesport and other sports.

Variables	Dancesport (*n* = 21)	Other Sports (*n* = 27)	*p* *
**Age** (years), mean ± SD (range)	54.6 ± 13.9 (29–73)	47.4 ± 11.1 (20–70)	NS
**Sex**, *n* (%)			
Male	10 (47.6)	19 (70.4)	NS
Female	11 (52.4)	8 (29.6)	
**Blindness**, *n* (%)			
Congenital	13 (61.9)	16 (59.3)	NS
Acquired	8 (38.1)	11 (40.7)	
**Visual disability level**, *n* (%)			
Blind	14 (66.7)	23 (85.2)	NS
Severely sight-impaired	7 (33.3)	3 (11.1)	
Mildly sight-impaired	0 (0)	1 (3.7)	
**Educational level**, *n* (%)			
Primary school degree	1 (4.8)	0 (0)	NS
Middle school degree	3 (14.3)	1 (3.7)	
High school degree	13 (61.9)	13 (48.15)	
University	4 (19.0)	13 (48.15)	

NS, not significant; SD, standard deviation. * Unpaired Student’s *t*-test for the comparison of group means or chi-square for categorical variables.

**Table 2:** Mean scores of psychological well-being scale and quality of life questionnaire in visually impaired dancers compared with visually impaired athletes practicing different sports.

Variables	Dance Mean ± SD (SEM)	Other Sports Mean ± SD (SEM)	*t*	df	*p* **
**Psychological well-being** #					
Autonomy	12.9 ± 1.20 (0.263)	11.70 ± 2.14 (0.413)	2.388	46	0.021
Environmental mastery	12.29 ± 2.05 (0.448)	10.52 ± 2.33 (0.448)	2.746	46	0.009
Personal growth	13.19 ± 2.06 (0.450)	13.04 ± 1.95 (0.375)	0.264	46	0.793
Positive relations with others	9.57 ± 2.84 (0.619)	8.44 ± 2.15 (0.415)	1.565	46	0.124
Purpose in life	10.86 ± 3.65 (0.797)	10.11 ± 2.66 (0.513)	0.819	46	0.417
Self-acceptance	13.48 ± 2.79 (0.608)	11.30 ± 2.76 (0.531)	2.705	46	0.010
Total score	72.33 ± 8.29 (1.810)	65.11 ± 5.91 (1.138)	3.522	46	<0.001
**Quality of life** *					
Physical	54.47 ± 4.27 (0.932)	51.57 ± 5.36 (1.031)	2.027	46	0.048
Mental	51.83 ± 8.36 (1.824)	46.49 ± 8.85 (1.703)	2.127	46	0.039

SD, standard deviation; SEM, standard error of the mean; df, degrees of freedom. # Assessed by 18-item Psychological Well-Being (PWB-18) scale. * Assessed by Short Form-12 (SF-12) questionnaire. ** Unpaired Student’s *t*-test.

**Table 3:** Multiple linear regression analysis of characteristics of visually impaired athletes to predict psychological well-being.

Variables	Psychological Well-Being Total Score			
	Coefficient	SE	*t*	*p*
Sex	–0.965	2.319	–0.416	0.679
Blindness	–1.984	2.244	–0.884	0.382
Visual disability level	–3.026	2.302	–1.315	0.196
Educational level	–1.870	1.615	–1.158	0.254
Years of sport practice	–0.161	1.471	–0.110	0.913
Practiced sport	6.070	2.620	2.317	0.026

SE, standard error.

**Table 4:** Multiple linear regression analysis of characteristics of visually impaired athletes to predict physical quality of life.

Variables	Quality of Life Physical			
	Coefficient	SE	*t*	*p*
Sex	1.101	1.533	0.719	0.476
Blindness	0.760	1.484	0.513	0.611
Visual disability level	–0.533	1.522	–0.350	0.728
Educational level	3.110	1.067	2.913	0.006
Years of sport practice	0.103	0.972	–0.351	0.916
Practiced sport	5.560	1.732	3.210	0.003

SE, standard error.

**Table 5:** Multiple linear regression analysis of characteristics of visually impaired athletes to predict mental quality of life.

Variables	Quality of Life Mental			
	Coefficient	SE	*t*	*p*
Sex	3.824	2.833	1.350	0.185
Blindness	4.161	2.743	1.517	0.137
Visual disability level	1.065	2.813	0.379	0.707
Educational level	0.981	1.973	0.497	0.622
Years of sport practice	1.555	1.798	0.865	0.392
Practiced sport	7530	3.202	2.352	0.024

SE, standard error.

## Data Availability

All relevant data are included within the manuscript.

## Supplementary Materials

The following supporting information can be downloaded at: https://www.mdpi.com/article/10.3390/ijerph19084438/s1, English version of the administered questionnaire.

## References

[1] Li Q.D., Kuang X.M., Qi J. (2020). Correlates of physical activity of children and adolescents with visual impairments: A systematic review. Curr. Pharm. Des..

[2] Sweeting J., Merom D., Astuti P.A.S., Antoun M., Edwards K., Ding D. (2020). Physical activity interventions for adults who are visually impaired: A systematic review and meta-analysis. BMJ Open.

[3] Kamelska A.M., Mazurek K. (2015). The assessment of the quality of life in visually impaired people with different level of physical activity. Phys. Cult. Sport. Stud. Res..

[4] Cheikh Y., Zenagui S. (2019). The effect of a physical fitness program on the health-related physical fitness components of blind male students. Gazz. Med. Ital.-Arch. Sci. Med..

[5] Ilhan B., Idil A., Ilhan I. (2021). Sports participation and quality of life in individuals with visual impairment. Ir. J. Med. Sci..

[6] Warburton D.E.R., Bredin S.S.D. (2017). Health benefits of physical activity: A systematic review of current systematic reviews. Curr. Opin. Cardiol..

[7] Kim J., Park S.-H. (2018). Leisure and health benefits among Korean adolescents with visual impairments. Int. J. Qual. Stud. Health Well-Being.

[8] van der Ploeg H.P., van der Beek A.J., van der Woude L.H.V., van Mechelen W. (2004). Physical activity for people with a disability: A conceptual model. Sports Med..

[9] Lee M., Zhu W., Ackley-Holbrook E., Brower D.G., McMurray B. (2014). Calibration and validation of the physical activity barrier scale for persons who are blind or visually impaired. Disabil. Health J..

[10] Bell L., Wagels L., Neuschaefer-Rube C., Fels J., Gur R.E., Konrad K. (2019). The cross-modal effects of sensory deprivation on spatial and temporal processes in vision and audition: A systematic review on behavioral and neuroimaging research since 2000. Neural Plast..

[11] Bertonati G., Amadeo M.B., Campus C., Gori M. (2021). Auditory speed processing in sighted and blind individuals. PLoS ONE.

[12] Gori M., Amadeo M.B., Campus C. (2020). Spatial metric in blindness: Behavioural and cortical processing. Neurosci. Biobehav. Rev..

[13] Haibach P.S., Wagner M.O., Lieberman L.J. (2014). Determinants of gross motor skill performance in children with visual impairments. Res. Dev. Disabil..

[14] Alotaibi A.Z., Alghadir A., Iqbal Z.A., Anwer S. (2016). Effect of absence of vision on posture. J. Phys. Ther. Sci..

[15] Pigeon C., Li T., Moreau F., Pradel G., Marin-Lamellet C. (2018). Cognitive load of walking in people who are blind: Subjective and objective measures for assessment. Gait Posture.

[16] Pereira R.C.M., Vigário P.S., Mainenti M.R.M., Silva D.T.R., Lima T.R.L., Lemos T. (2019). Computerized photogrammetric assessment of postural alignment in visually impaired athletes. J. Bodyw. Mov. Ther..

[17] da Silva E.S., Fischer G., da Rosa R.G., Schons P., Teixeira L.B.T., Hoogkamer W., Peyré-Tartaruga L.A. (2018). Gait and functionality of individuals with visual impairment who participate in sports. Gait Posture.

[18] Foisy A., Kapoula Z. (2017). Plantar Exteroceptive Inefficiency causes an asynergic use of plantar and visual afferents for postural control: Best means of remediation. Brain Behav..

[19] Rogge A.K., Hamacher D., Cappagli G., Kuhne L., Hötting K., Zech A., Gori M., Röder B. (2021). Balance, gait, and navigation performance are related to physical exercise in blind and visually impaired children and adolescents. Exp. Brain Res..

[20] Serin-Brackman V., Pezet Poux J., Quintyn J.C. (2019). Postural changes in patients with visual deficits. J. Fr. Ophtalmol..

[21] Russo M.M., Lemos T., Imbiriba L.A., Ribeiro N.L., Vargas C.D. (2017). Beyond deficit or compensation: New insights on postural control after long-term total visual loss. Exp. Brain Res..

[22] Gori M., Campus C., Signorini S., Rivara E., Bremner A.J. (2021). Multisensory spatial perception in visually impaired infants. Curr. Biol..

[23] Cattaneo Z., Vecchi T., Cornoldi C., Mammarella I., Bonino D., Ricciardi E., Pietrini P. (2008). Imagery and spatial processes in blindness and visual impairment. Neurosci. Biobehav. Rev..

[24] Pasqualotto A., Proulx M.J. (2012). The role of visual experience for the neural basis of spatial cognition. Neurosci. Biobehav. Rev..

[25] Després O., Candas V., Dufour A. (2005). The extent of visual deficit and auditory spatial compensation: Evidence from self-positioning from auditory cues. Cogn. Brain Res..

[26] Seemungal B.M. (2015). The components of vestibular cognition—Motion versus spatial perception. Multisens. Res..

[27] Sukemiya H., Nakamizo S., Ono H. (2008). Location of the auditory egocentre in the blind and normally sighted. Perception.

[28] Vercillo T., Tonelli A., Gori M. (2018). Early visual deprivation prompts the use of body-centered frames of reference for auditory localization. Cognition.

[29] Mirandola D., Monaci M., Miccinesi G., Vannuzzi A., Sgambati E., Manetti M., Marini M. (2019). Psychological well-being and quality of life in visually impaired baseball players: An Italian national survey. PLoS ONE.

[30] Bahrick L.E., Lickliter R., Castellanos I., Todd J.T. (2015). Intrasensory redundancy facilitates infant detection of tempo: Extending predictions of the intersensory redundancy hypothesis. Infancy.

[31] Cappagli G., Finocchietti S., Cocchi E., Giammari G., Zumiani R., Cuppone A.V., Baud-Bovy G., Gori M. (2019). Audio motor training improves mobility and spatial cognition in visually impaired children. Sci. Rep..

[32] Larsson L., Frandin K. (2006). Body awareness and dance—Based training for persons with acquired blindness-Effect on balance and gait speed. Vis. Impair. Res..

[33] Mavrovouniotis F.I., Papaioannou C.S., Argiriadou E.A., Mountakis C.M., Konstantinakos P.D., Pikoula I.T., Mavrovounioti C.F. (2013). The effect of a combined training program with Greek dances and Pilates on the balance of blind children. J. Phys. Educ. Sport.

[34] Laird K.T., Vergeer I., Hennelly S.E., Siddarth P. (2021). Conscious dance: Perceived benefits and psychological well-being of participants. Complement. Ther. Clin. Pract..

[35] Federici A., Bellagamaba S., Rocchi M.B. (2005). Does dance-based training improve balance in adult and young old subjects? A pilot randomized controlled trial. Aging Clin. Exp. Res..

[36] Ardakani M.K., Shalamzari M.H., Mansori M.H. (2020). Effect of core stability training on postural control, risk of falling, and function of the blind: A randomized controlled trial. Balt. J. Health Phys. Act..

[37] Kreutz G. (2008). Does partnered dance promote health? The case of tango Argentino. J. R. Soc. Promot. Health.

[38] Fancourt D., Finn S. (2019). What Is the Evidence on the Role of the Arts in Improving Health and Well-Being? A Scoping Review.

[39] Onutu A.H., Rus C., Acalovschi I. (2017). The public perception of the anaesthesiologist in Romania: A survey. Rom. J. Anaesth. Intensive Care.

[40] Kilanowski J.F. (2018). Using Google to Survey PNPs About Agricultural Safety. J. Pediatr. Health Care.

[41] Ruini C., Ottolini F., Rafanelli C., Ryff C., Fava G.A. (2003). Italian validation of psychological well-being Scales (PWB). Riv. Psichiatr..

[42] Picardi A., Bartone P.T., Querci R., Bitetti D., Tarsitani L., Roselli V., Maraone A., Fabi E., De Michele F., Gaviano I. (2012). Development and validation of the Italian version of the 15-item dispositional resilience scale. Riv. Psichiatr..

[43] Sagone E., De Caroli M.E. (2014). Relationships between psychological well-being and resilience in middle and late adolescents. Procedia-Soc. Behav. Sci..

[44] Apolone G., Mosconi P., Quattrociocchi L., Gianicolo E., Groth N., Ware J.E. (2001). Questionario Sullo Stato di Salute SF-12.

[45] Marini M., Monaci M., Manetti M., Piazza M., Paternostro F., Sgambati E. (2015). Can practice of Dancesport as physical activity be associated with the concept of “successful aging”?. J. Sports Med. Phys. Fitness.

[46] Ryff C.D. (1989). Happiness is everything, or is it?. Explorations on the meaning of psychological well-being. J. Pers. Soc. Psychol..

[47] Ryff C.D., Keyes C.L.M. (1995). The structure of psychological well-being revisited. J. Pers Soc. Psychol..

[48] Varma R., Wu J., Chong K., Azen S.P., Hays R.D., Los Angeles Latino Eye Study Group (2006). Impact of severity and bilaterality of visual impairment on health-related quality of life. Ophthalmology.

[49] Kaplan R.M., Ries A.L. (2007). Quality of life: Concept and definition. COPD J. Chronic Obstr. Pulm. Dis..

[50] Doré I., Caron J. (2017). Mental Health: Concepts, Measures, Determinants. Sante Ment. Que..

[51] Wallin N.L., Merker B., Brown S. (2000). The Origins of Music.

[52] Miller G. (2000). Evolution of human music through sexual selection. The Origins of Music.

[53] Miller G. (2001). Aesthetic fitness: How sexsual selection shaped artistic virtuosity as a fitness indicator and aesthetic preferences as mate choice criteria. Bull. Psychol. Arts.

[54] Bergstein C.D. (2010). Young children and movement: The power of creative dance. YC Young Child..

[55] Fong Yan A., Cobley S., Chan C., Pappas E., Nicholson L.L., Ward R.E., Murdoch R.E., Gu Y., Trevor B.L., Vassallo A.J. (2018). The effectiveness of dance interventions on physical health outcomes compared to other forms of physical activity: A systematic review and meta-analysis. Sports Med..

[56] Quiroga Murcia C., Kreutz G., Clift S., Bongard S. (2010). Shall we dance? An exploration of the perceived benefits of dancing on well-being. Arts Health.

[57] Lahav O., Mioduser D. (2008). Construction of cognitive maps of unknown spaces using a multi-sensory virtual environment for people who are blind. Comput. Hum. Behav..

[58] Schaffert N., Janzen T.B., Mattes K., Thaut M.H. (2019). A review on the relationship between sound and movement in sports and rehabilitation. Front. Psychol..

[59] Koch S.C., Riege R.F.F., Tisborn K., Biondo J., Martin L., Beelmann A. (2019). Effects of dance movement therapy and dance on health-related psychological outcomes. A meta-analysis update. Front. Psychol..

[60] Homann K.B. (2010). Embodied concepts of neurobiology in dance/movement therapy practice. Am. J. Dance Ther..

[61] Thaut M.H., McIntosh G.C., Hoemberg V. (2015). Neurobiological foundations of neurologic music therapy: Rhythmic entrainment and the motor system. Front. Psychol..

[62] Crasta J.E., Thaut M.H., Anderson C.W., Davies P.L., Gavin W.J. (2018). Auditory priming improves neural synchronization in auditory-motor entrainment. Neuropsychologia.

