# Supplementary material for: Survey on Psychological Well-Being and Quality of Life in Visually Impaired Individuals: Dancesport vs. Other Sound Input-Based Sports
Source: Int J Environ Res Public Health. 2022 Apr 7;19(8):4438. doi: 10.3390/ijerph19084438 (PMC9024582; doi:10.3390/ijerph19084438)
Supplement: Supplementary file 1 [file ijerph-19-04438-s001.zip › ijerph-1665329-supplementary.pdf]

Questionnaire

First name___________________

Surname initial letter________________

Gender ☐ male ☐ female

Date of birth ________________

Educational level?

- ☐ No study
- ☐ Primary school
- ☐ Middle school
- ☐ High school
- ☐ University

Your visual impairment is?

- ☐ Congenital
- ☐ Acquired

Your visual impairment classification?

- ☐ Blind
- ☐ Severely sight-impaired
- ☐ Mildly sight-impaired

Are you currently practicing a sport activity?

- ☐ Yes
- ☐ No

If you answered yes, which sport?

- ☐ Futsal
- ☐ Showdown
- ☐ Dancesport
- ☐ Tennis
- ☐ Baseball

How long have you been playing your sport activity?

- ☐ Six months to one year
- ☐ One to three years
- ☐ Over three years

Name of your sport association/team?

____

Have you ever been diagnosed with following diseases?

- ☐ Behavioral/mental disorders
- ☐ Chronic pathologies
- ☐ No disease

PWB-18 SCALE

Read each question and sign carefully to which extent each statement is proper for you. Using a scale of 1 (strongly disagree) to 6 (strongly agree).

	Strongly disagree 1	2	3	4	5	Strongly agree 6
1. I tend to be influenced by people who have strong personality						
2. I have confidence in my own opinions even if they are opposed to those of others						
3. I judge myself by what I think is important and not by what others think is important						
4. In general, I feel I am in charge of the situation in which I live						
5. The requests of everyday life often bring me down						
6. I am very good at managing the many responsibilities of everyday life						
7. I think it is important to have new experiences that help you to compare your opinion of yourself and the world around you						
8. For me life has been a continuous process of learning, change and growth						
9. I gave up trying to make big improvements or changes in my life a long time ago						
10. For me has been difficult and frustrating to maintain stable relationships						
11. People would describe me as an available person, ready to share my time with others						
12. I have not experienced many warm and trusting relationships with others						
13. I live life one day at a time and don't really think about the future						
14. Some people have no plans, but I am not one of them						
15. Sometimes I have the feeling that I have already done everything that could be done						
16. I am pleased with how things have turned out in my life						
17. I like most aspects of my personality						
18. Many times I am not satisfied with the results achieved in my life						

SF-12

Read each question and sign carefully which statement is proper for you.

1. In general, would you say your health is?
Excellent.......................... 1
Very Good.................................. 2
Good.. 3
Fair.. 4
Poor.. 5

2. Does your health now limit you during moderate physical activities (such as moving a table, pushing a vacuum cleaner, bowling or cycling, etc.)?
Yes, limited a lot............ 1
Yes, limited a little................ 2
No, not limited at all..................... 3

3. Does your health now limit you during climbing a few flights of stairs?
Yes, limited a lot............ 1
Yes, limited a little................ 2
No, not limited at all..................... 3

4. During the past 4 weeks, have you accomplished less than you would like with your work or other regular daily activities as a result of your physical health?
Yes............ 1
No.................... 2

5. During the past 4 weeks, did you have to limit certain types of work or other activities as a result of your physical health?
Yes............ 1
No 2

6. During the past 4 weeks, have you accomplished less than you would like with your work or other regular daily activities as a result of any emotional problem (such as feeling depressed or anxious)?
Yes............ 1
No 2

7. During the past 4 weeks, did you have a concentration decline at work or in other activities as a result of any emotional problem (such as feeling depressed or anxious)?
Yes............ 1
No.................... 2

8. During the past 4 weeks, how much did pain interfere with your normal work (including both work outside home and housework)?
Not at all............ 1
A little bit................. 2
Moderately.............................. 3
Quite a bit 4
Extremely.................................. 5

9. How much of the time during the past 4 weeks, have you felt calm and peaceful?

All of the time 1
Most of the time.......... 2
A good bit of the time 3
Some of the time............ 4
A little of the time................................ 5
None of the time 6

10. How much of the time during the past 4 weeks, have you felt full of energy?

All of the time 1
Most of the time.......... 2
A good bit of the time 3
Some of the time............ 4
A little of the time................................ 5
None of the time 6

11. How much of the time during the past 4 weeks, have you felt downhearted and sad?

All of the time 1
Most of the time.......... 2
A good bit of the time 3
Some of the time............. 4
A little of the time... 5
None of the time 6

12. During the past 4 weeks, how much of the time has your physical health or emotional status interfered with your social activities (for example with friends, relatives, etc.)?

All of the time 1
Most of the time.......... 2
A good bit of the time 3
Some of the time............ 4
A little of the time................................ 5
None of the time 6
